# Contributions to the Knowledge of Diseases of the Skin

**Published:** 1842-04

**Authors:** 


### Art. XIII.

*Bidrag til Kundskab af de endemiske Hudsygdomme.* Af Brigadelæge Dr. Hjort. Förste Stykke.—*Christiania,* 1840. 8vo, pp. 25.

*Contributions to the Knowledge of Endemic Diseases of the Skin.* By Dr. Hjort, Brigade-physician in Christiania.—*Christiania,* 1840. (*Reprinted from the "Norsk Magazin for Lægevidenskaben."* Vol. I. No. I. 1840.)

This is the first of a series of papers on the diseases of the skin endemic in Norway, the investigation of which has been undertaken by Dr. Hjort. The essay does infinite credit to the author; and as it gives a view of an ill-understood disease very different from any heretofore communicated to the profession, and we doubt not, much more accurate, we shall transfer the greater portion of its contents to our pages, in the form of an analysis, without any comment. On a future occasion when we have had all Dr. Hjort's views and opinions before us, we may enter into a more critical examination of the subject. The object of the author in the present essay, is to give a more precise and complete description of the radesyge, or Norwegian leprosy, than has been before attempted; little or nothing being said on its pathology and treatment. The disease, as is well known, is of frequent occurrence in Norway, but is rarely to be met with south of the Baltic. The author has for the last seventeen years been engaged in the close study of the radesyge, as it occurs in the provinces and capital of Norway, and from having held for several years the post of assistant-physician to the hospital for skin diseases in Christiania, he has enjoyed peculiar facilities for contemplating this disease. Lately also, in compliance with the wishes of the Norwegian government, he has visited many parts of central and of southern Europe, for the purpose of making himself acquainted with those maladies which have been by some writers considered as closely allied to radesyge, and by others have been actually identified with that affection. The result of this uncertainty has been, that several very different disorders have been confounded together under the common name of radesyge; syphilis, elephantiasis græcorum, scrofula, and scurvy, having been all at one time or other described under that appellation. Amid such a confusion of terms and symptoms, Dr. Hjort has ventured to coin a new name for this disorder, and adopted from Hippocrates the term Thæria,* from θηριον (ελκος) a malignant ulcer. The term lepra has been already applied to three or four different diseases, and as that of radesyge is merely provincial, he proposes to raise this disorder to the rank it merits in nosology, by affixing to it this classical appellation.

General symptoms of radesyge (thæria). The individuals affected are usually of the inferior class, and after their health has for a longer or shorter period been declining without any marked symptoms to localize the disease, the skin, and occasionally the subjacent cellular tissue, is attacked with inflammation, which is chronic in its progress, and tends invariably to suppuration, after which, the pain before experienced either altogether ceases or at least is greatly alleviated. At the first appearance of the inflammatory symptoms above alluded to, there is formed an in-

* We follow the orthography of the author, although the Latin derivative of θηριον, according to the usual rule, should be *Theria* not *Thæria.*

durated, ill-defined, blueish-red tumour in the substance of the skin, which after a short time softens and forms an ulcer of irregular shape, with rounded edges, and bordered by a diffuse redness, shaded with blue and gray, and passing from a rose-red to a violet or a lead-gray colour. The surface of the sore is unequal and granulating, and secretes a greenish-yellow inodorous pus, which when exposed to the atmosphere, concretes into hard, yellowish-green or blackish-green scabs. After the ulcerative process has destroyed the affected portion of the skin, and perhaps also the subjacent cellular tissue, healthy granulations push forth from the bottom of the sore, and these at length form a somewhat irregular, but very firm cicatrix. In the mean time new tumours have appeared in other parts of the skin, and these sooner or later run a similar course. In this manner the disease, when left to itself, proceeds, and may continue for months or even years without causing any serious injury to the patient, except in cases where it attacks the nose or pharynx, or where necrosis is caused by periostitis on the adjacent bones.

It is not more than a hundred years since this malady was first noticed in Norway and Sweden, and it has since then been detected on the Baltic coasts and on the northern coast of the Adriatic.

*Premonitory symptoms.* The outbreak of the disease is almost always preceded by catarrhal, rheumatic, or nervous affections, whilst the intellect is obscured, the individual becoming in a marked manner more dull and obtuse than before. But the most decided preliminary symptoms are flying pains in the limbs, which however are seldom so severe as to disturb the sleep of the patient, or to prevent his continuing his daily labour. Such pains often concentrate themselves in certain parts and organs of the body, as in the head under the type of hemicrania, or as pain darting from the auditory passages to the neck, or as pains in the shin-bone and forearm, where they are generally accompanied with swelling of the superficial portions of the tibia and ulna. Rarely are they observed to increase at night; on the contrary, they often disappear when the patient is warm in bed. In general they cease altogether, or at least greatly diminish in intensity when the local affection breaks out in some part or other of the skin; and such local affections usually appear in the neighbourhood of those parts where such pains have previously concentrated themselves: thus, after hemicrania, ulcers form upon the head; and sores show themselves over the tibia and ulna after pains in the leg and forearm. But to this general rule there are many exceptions, the skin being often affected only in a single spot after flying pains had pervaded the whole body. The pains in the tibia and ulna are, among all the chronic affections preceding the outbreak of radesyge, those which merit the most attention. After the patient has for some time complained of lancinating pains in the bone, an even, hard swelling presents itself on its most superficial portion, which may remain stationary for years without any alteration in the structure or colour of the skin. But when the ulceration peculiar to the disease has shown itself in any part of the body, the pains in the periosteum generally subside, although the above-mentioned enlargement of the bone continues throughout life. When the skin covering the superficial portion of the tibia is attacked by the disorder, the subjacent bone is sometimes affected with necrosis, but this is by no means connected with the superficial elevation of the bone before alluded to.

The first symptoms of radesyge often appear as sequelæ of acute disorders, such as rheumatic fever, bronchitis, pleurisy, &c. But these maladies must not be looked on as proximate causes, but merely as eliminating the germ of the disease previously existing in the body. Occasionally Dr. Hjort has observed the ulcers of radesyge to vanish at the commencement of an acute attack, and to reappear on the termination of the attack.

Particular symptoms of radesyge. Although the disorder consists principally of the above-described inflammation and ulceration of the skin and of its subjacent cellular tissue, yet this morbid process varies so much in its extent, seat, and progress, that it is necessary to specify more at length the various forms under which this remarkable disease presents itself. For this purpose the author divides the malady into two great groups, according to the symptoms, namely, 1st, Affections of the external skin; 2d, Affections of the internal skin or mucous membrane.

*A.* Affections of the external skin. The principal seat of the disease is always the deepest layer of the skin (corium), but the subjacent cellular tissue is usually more or less affected. Upon the amount to which this tissue participates in the disease, depends much the appearance which it assumes. In the most superficial forms of the disorder, the skin and the cellular tissue in its immediate neighbourhood are alone affected, and thereby it so nearly approaches in character to a pustular disease, that the author has proposed for this form the name of *thæria pustulosa;* or, rather, *pustuloides*, as real pustules do not actually appear, but small abscesses supply their place. In a second form of radesyge, the subcutaneous cellular tissue is involved in its whole thickness, and then forms tubercles beneath the skin. This Dr. Hjort names *thæria tuberculosa*. The third variety he names *thæria phlegmonosa*, as in this variety the skin and cellular tissue to a great depth are affected, so as to cause much swelling of the parts involved, but the surface of such tumours are seldom uneven as in the tubercular form of the disease.

1. *Thæria pustulosa, seu cutanea.* Its distinguishing characteristic is a round ulcer of the size of a pea, which gradually destroys the whole thickness of the skin, and is covered with a firm and dense scab. When left to themselves, these ulcers may require weeks or months to run their complete course to cicatrization. Occasionally slight itching and pain reveal the presence of these pustules; but they often exist unnoticed, till discovered by the eye of the patient or of his medical attendant. The entire thickness of the skin at first assumes a blueish red colour, and though not elevated above the surrounding healthy tissue, it is somewhat harder to the touch. A single point in this injected portion now begins to soften, but yet it does not raise the superincumbent skin after the manner of an abscess; the presence of pus is only revealed by an indistinct fluctuation. The skin above this small abscess becomes thinner, and at the same time shrivels up in the centre till it becomes a hard firm scab, which constantly increases in thickness over the subjacent purulent matter. Fresh healthy granulations now begin to shoot forth from the bottom of the abscess, and these form, with the scab that still remains, a firm cicatrix, after which the scab or crust falls off. According to the mode in which these pustules are grouped together, Dr. Hjort has bestowed upon this form the names of thæria pustulosa sparsa, thæria pustulosa figurata, thæria pustulosa area.

1. In the first variety, *thæria pust. sparsa,* the ulcers are solitary, and occur chiefly on the head and back.

2. *Thæria p. figurata* is the most frequent form, and the pustules are here grouped as in impetigo figurata. Such groups are to be observed on the forehead or cheeks, and still more frequently on the back, breast, or on the extremities in the neighbourhood of the joints. These patches vary in extent from two to six or eight inches; their form is irregular, never presenting the distinct circles of the serpiginous syphilitic eruptions. The pustules make their appearance close to each other, and spread outwards as from a common centre, yet occasionally pustules are developed in parts that have already been affected. The cicatrices are firm, white, and uneven, their surface being traversed by numerous white bands, whilst their circumference is so irregular as to have been compared by Rust, of Berlin, to land on a map surrounded by sea.

3. *Thæria pust. area.* In this rare form of the disease, and of which Dr. Hjort has seen but five or six examples, the skin is infiltrated in one continuous extent of several inches in diameter. When so affected it is of a deep blueish, red, or lead-gray hue, and though hard as wood to the touch, is not sensibly elevated above the surrounding healthy skin. Upon this surface pustules subsequently appear, which remain perfectly distinct one from the other, and leave behind them small round scars, which appear like drops of milk scattered upon the skin after it has returned to its healthy state. This variety has been chiefly observed upon the loins, buttocks, and forearms.

II. *Thæria tuberculosa seu subcutanea.* This form is by far the most frequent of all, and may serve as a prototype of the disease. It first exhibits itself in the form of tubercles, which are elevated two or three lines above the surface of the skin, and are in general of a dirty-white or lead-gray colour, and somewhat harder than the surrounding healthy tissue, but their colour remains sometimes unaltered until the process of ulceration has commenced. The diameter of these tubercles varies from that of a hazelnut to that of a walnut, their form is generally round, yet their circumference is never accurately defined. Such tubercles may exist unaltered for weeks or months, and then begin to soften from their centre, the superincumbent skin becoming daily thinner, till an ulcer appears, which seldom attains the diameter of more than half an inch. These ulcers are somewhat rounded in form, but never present the circular appearance of syphilitic sores, and their borders appear obtuse, though they may at the same time be deeply undermined. These ulcers are from two to three lines in depth, and their surface secretes the peculiar yellowish-green pus which, when exposed to the air, concretes into hard black scabs. The cicatrix is gradually formed by fresh granulations shooting from the surface of the ulcer. The tubercles of radesyge are seldom solitary, but generally in groups, where they may be observed in various stages of development at the same time, which gives to the skin in the parts thus affected a most irregular and variegated appearance. This form of the disease is frequent on all parts of the body excepting the palms of the hands and soles of the feet, but is most commonly observed on the loins and extremities, and particularly (on the anterior surface of the tibia) from the knee to the ankle.

III. *Thæria phlegmonosa seu exedens.* The hitherto-described forms of Radesyge have their seat chiefly in those parts of the skin which lie contiguous to bones, tendons, or ligaments; but this variety appears by preference in the most fleshy parts of the body, as in the cheeks, the mammæ, the nates, or the forearm. In such situations, the skin along with the entire subjacent cellular tissue swells up for the space of several inches round, the tumefied part being in general hard and resistent to the touch, but not painful on pressure, and the colour remains constantly of a deep blueish-red. The ulcers formed in this variety are often many inches in circumference, their surface is extremely irregular, and secretes a greenish-yellow inodorous pus, which concretes into the usual scabs on the edges of the sore, while the copious secretion from the centre prevents there the formation of the usual crust. In the mean time large portions of cellular tissue are thrown off by suppuration, and occasionally this morbid process insinuates itself between the muscles and ligaments, leaving them bare and as it were dissected at the bottom of the wound. The cicatrices of these large ulcers are naturally deep and irregular. By careful treatment they may occasionally, after having remained open for years, be closed in the space of a few weeks, but the cicatrix is then peculiarly deep, from the rapid contraction of the skin in healing. This form of the malady often attacks the whole leg from the knee to the ankle, and the limb then presents a most remarkable appearance, not a little resembling that of a limb affected with elephantiasis, from the innumerable hard elevations that form in the cellular tissue beneath the skin, and which are often of the size of a hen's egg or more, whilst the skin retains its normal colour and consistence, except that it is distended by the exceedingly hard tumours beneath.

Dr. Hjort here speaks of the mode in which the osseous system is effected in radesyge. In some instances the pains in the long and flat bones do not remit on the appearance of the ulcerative process in the skin. The tumefaction of the periosteum increases, and this membrane is gradually detached from the subjacent bone, and fluctuation is perceptible in the centre of the tumour; the skin, at first natural in colour, gradually becomes of a blueish-red hue, ulcerates, and at the bottom of the sore the bone appears denuded of its periosteum, and a considerable portion of it is frequently detached by necrosis. In general the long bones are affected simultaneously with the skin and cellular membrane. This often occurs in the second form of the disease (thæria tuberculosa), the periosteum becoming affected where it lies nearly in contact with the skin, as on the forehead, on the zygomatic arch, on the cheek, or on the ulnar portion of the forearm. The caries that ensues is quite superficial, terminating with the loss of a few thin lamellæ of bone.

*B.* Affections of the mucous membranes. Within the body, the disease attacks the mucous membranes lining the nose, the mouth, and the pharynx; it has rarely extended itself to the air-passages. The progress of the disorder is similar to that in the external skin, except that it is somewhat modified by the peculiar properties of the mucous membrane in different parts. Under the titles of, 1, Thæria faucium; 2, Thæria cavitatis oris; 3, Thæria cavitatis nasi,—Dr. Hjort gives a full description of these peculiarities, of which the following are the principal features:

*Thæria faucium.* Next to the external skin, the fauces are, of all parts of the body, the most liable to be attacked by the radesyge. Out of 368 individuals labouring under this disease, 125 suffered from the affection of the fauces, and the site of the disorder was chiefly in the uvula and in the velum pendulum palati. In a few cases the progress of the disorder was subacute; the velum pendulum palati and uvula swelled and became of a dark violet erysipelatous hue, and the patient complained of some obstruction in deglutition, but remained free from fever or disorder of the gastric organs. After the lapse of two or three weeks, a yellow point showed itself in this part, as the commencement of ulceration, which then rapidly advanced, till it destroyed the uvula or perforated the velum. The ulcerations thus formed are of irregular shape, and uneven surface, and from the latter is secreted a yellowish green pus. They resemble not a little the abscesses which form in the throat from quinsy, but in radesgye the ulceration continues to spread, unless checked by appropriate treatment, until it has destroyed the whole or a large part of the pendulous palate. The disorder having run the above described course, cicatrization takes place by granulations shooting from the surface of the sore; the purulent matter diminishes, till merely a colourless mucus is secreted; and lastly, there is formed a firm but extremely irregular cicatrix. The course of the disease is sometimes absolutely chronic, the symptoms being as above described, but requiring months or years to accomplish that which occurs in a few weeks in the subacute form of the disorder. The colour of the affected portion is in such cases a blueish-red, and the pain so slight that the patient is not aware of the existence of the disease in the fauces, till a great loss of substance materially affects either the speech or the deglutition. The mucous membrane of the pharynx is seldom affected unless the disorder prevail at the same time in the pendulous palate. The appearances in the pharynx are similar to those above described, speech and deglutition are of course much affected, and occasionally Dr. Hjort has seen caries of the vertebræ of the neck supervene from extension of the disorder to their periosteum.

2. *Thæria cavitatis oris.* The hard palate is here the part most generally affected, but in rare instances the disease has been observed upon the tongue, and once only on the inner surface of the cheeks. The course of this form of the malady does not differ from that above described, but the periosteum always suffers in the affection of the hard palate, and portions of the bones are frequently separated by necrosis, so as to leave a passage open between the mouth and nose. The disease shows itself in the hard palate as a firm blueish swelling, which after a considerable period runs on to ulceration. When the bony portions have come away, the ulcer often heals, but imperfectly, so that a cavity, or even a false passage to the nose remains. This is also covered with fresh mucous membrane, and as the patients sometimes do not seek assistance till the malady has advanced thus far, a medical man might easily believe such an opening to be congenital. Dr. Hjort has seen one case where a fistulous opening, whose diameter did not exceed that of a mustard seed, passed quite through to the nasal cavity. In rare instances, the malady has attacked the alveoli of the teeth.

3. *Thæria cavitatis nasi.* In this variety the ulceration has its seat in and usually perforates the septum narium, and occasionally the end of the nose sinks in from complete destruction of that part. It has also been observed to affect a portion of the Schneiderian membrane covering the septum osseum, and to cause necrosis of the bones of that part. In 368 cases of radesyge this form of the disorder occurred in 73.

Finally, Dr. Hjort maintains that the above-described disorders are to be considered as one and the same malady; and this assertion is based upon the following grounds:

1. These various forms of disease have their seat in analogous tissues, namely, in the internal and external skin, and in the subjacent cellular membrane; and the affection of the osseous system occurs either primarily as a precursor of the radesyge, or secondarily in consequence of the extension of the cutaneous disorder.

2. The disease constantly tends to ulceration, which in all forms presents the peculiar characters of radesyge.

3. The course of these affections is generally chronic, and they are seldom accompanied with much pain; nor do they exercise any peculiar influence upon the constitution of the patient.

4. Several of the different forms of the disorder may be present at the same time in the same individual. Where a person has been frequently affected with the disease, the succeeding attacks may often appear under a different form from that under which the disease first manifested itself.

5. In all parts of the world where Dr. Hjort has observed this malady to be endemic, he has likewise met with all or a greater part of the various forms above described.

6. All varieties of radesyge arise from similar causes. The predisposing causes are a raw cold climate, and the excessive use of ill-cooked and unwholesome farinaceous food (meelspisen, i.e. puddings, &c.), whilst the immediate existing cause is almost always cold.

7. All varieties of radesyge yield to the same plan of treatment, and, according to Dr. Hjort, the specific remedy for this disease is corrosive sublimate.

---

